# Inaugural editorial: advancing preclinical and translational research of relevance to medicine

**DOI:** 10.1136/bmjos-2017-ined

**Published:** 2017-09-25

**Authors:** Emily S Sena

**Affiliations:** Deanery of Clinical Sciences, University of Edinburgh, Edinburgh, UK

It is my distinct honour to address you as the inaugural Editor-in-Chief of *BMJ Open Science. BMJ Open Science* is a new online peer-reviewed open access journal where we provide a platform to promote and engender trust in the research we publish and transparency in the processes we use to achieve this. Our scope is broad and covers the whole spectrum of preclinical and translational biomedical research that is close to human medicine. 

## Why another journal?

We have launched this journal to address some of the limitations in biomedical research widely described as impeding the efficiency with which preclinical data translates to improvements in human health. These include how we design our experiments, how we conduct them, the data and experiments we choose to include in our research reports, the overall quality and clarity of our research reports, the peer review process, and timely access to our research reports and their underlying data. What makes this journal different is that we have taken an innovative approach in order to challenge our collective bias against null and negative results; the desire to follow the status quo, which for many has culminated in very successful careers and the consequences of the desire to maintain a competitive edge in an outrageously competitive discipline. At *BMJ Open Science* we support preprints and replication studies, advocate for transparency of methods used, differentiate between exploratory and confirmatory studies, and will publish all sound experiments irrespective of their findings. Understanding what doesn’t work as well as what does work, will provide clear insights that will underscore the utility of preclinical evidence. We are also proponents of open science and information sharing, and have engaged with community led initiatives, such as the Open Science Framework, to eliminate access barriers to methods, data and information from the research that we publish.

*BMJ Open Science*  welcomes original research articles (including registered reports), study protocols, methods papers, data descriptor articles, reporting and conduct guidelines, review articles, and position papers. The research questions addressed must be sound, not necessarily novel, important and pertinent to human health in a preclinical setting.

*BMJ Open Science* plans to be synonymous with rigour, transparency and reproducibility in research, reporting and peer review. It is a great honour to lead and work with a group of outstanding scientists from Europe, China, North and South America and from a broad range of biomedical disciplines who will serve as members of the Editorial Board, and are committed to the mission and scope of the journal.

I hope that you will read our published articles, submit your research, and contribute to peer review and discussion. We commit to being an excellent source of scientific information and look forward to working with the preclinical and translational biomedical community to achieve our mission.  

